# Small molecule inhibition of ubiquitin C-terminal hydrolase L1 alters cell metabolism proteins and exerts anti- or pro-tumorigenic effects contingent upon chemosensitivity status in high grade serous ovarian cancer

**DOI:** 10.3389/fphar.2025.1547164

**Published:** 2025-02-26

**Authors:** Corinne Jansen, Julia McAdams, Chloe Kim, Payton De La Cruz, Angelica Salaverria, Nicholas A. DaSilva, Kathryn Grive, Nicole E. James

**Affiliations:** ^1^ Program in Women’s Oncology, Women and Infants Hospital, Providence, RI, United States; ^2^ Department of Obstetrics and Gynecology, Warren-Alpert Medical School of Brown University, Providence, RI, United States; ^3^ School of Public Health, Brown University, Providence, RI, United States; ^4^ Therapeutic Sciences Graduate Program, Brown University, Providence, RI, United States; ^5^ Division of Biology and Medicine, Proteomics Facility, Brown University, Providence, RI, United States

**Keywords:** high grade serous ovarian cancer (HGSOC), chemoresisitance, chemosensitivity, LDN-57444, Uchl1

## Abstract

High grade serous ovarian cancer (HGSOC) is the most lethal of all gynecologic malignancies in which the majority of patients eventually develop chemoresistant recurrent disease. Ubiquitin C-terminal hydrolase L1 (UCHL1) is a deubiquitinating enzyme canonically known for its involvement in neurodegeneration, but recently has been shown to play a key role in tumorigenesis. Furthermore, UCHL1 has garnered attention across a multitude of cancer subtypes as it has the ability to be targeted through small molecule inhibition. Therefore, the goal of this present study was to elucidate mechanistic consequences of small molecule UCHL1 inhibition in HGSOC. Comparative label-free proteomic analysis of HGSOC cell line, OVCAR8 revealed prominent changes in cell metabolism proteins upon treatment with UCHL1 small molecule inhibitor, LDN-57444. Further validation via Western blot analysis revealed that changes in cell metabolism proteins differed in matched chemosensitive versus chemoresistant HGSOC cells. Finally, cell viability analysis demonstrated that a combinatorial carboplatin and LDN-57444 blockade produced a promotion or conversely, inhibition of cell death, in chemoresistant, and chemosensitve HGSOC cells, respectively. This phenomenon was further corroborated by respective differences in activation levels of common tumor cell growth pathways STAT3, MAPK/ERK, and AKT in chemoresistant versus chemosensitive HGSOC cells. Overall, this investigation established that pharmacologic targeting of UCHL1 produces differential effects according to HGSOC chemosensitivity status.

## Introduction

High-grade serous ovarian cancer (HGSOC) is a highly aggressive disease and the most lethal of all gynecologic malignancies. In the United States alone, it is estimated that in 2024, there would be 19,680 new diagnoses of ovarian cancer and 12,740 deaths attributed to this disease (American Cancer Society, 2024). While a relatively rare cancer diagnosis, with a lifetime risk 1.3% and a 5-year survival of 46%, ovarian cancer remains the fifth leading cause of cancer-related deaths in female patients (Siegel et al., 2020). This high rate of mortality from an HGSOC diagnosis is driven by many patients presenting at a late stage, and despite responding well to frontline platinum-taxane based chemotherapy, the majority of these patients recur shortly following initial remission (Luvero et al., 2019). While strategies for ovarian cancer surveillance have been examined in the general and high-risk populations, at this time there are no diagnostic or therapeutic options that have been shown to change overall survival outcomes at this time, with the exception of patients that are homologous recombination deficient (Jacobs et al., 2016; Rosenthal et al., 2017). Therefore, novel targeted treatments are greatly needed for patients who do not respond to traditional chemotherapy-based approaches.

Ubiquitin C-terminal hydrolase-L1 (UCHL1) is a deubiquitinating enzyme that possesses dual activity of deubiquitination and ubiquitin (Ub) ligase activity (Liu et al., 2002). UCHL1 is involved in regulation of free Ub pools, lysosomal activity, cellular signaling and cytoskeleton dynamics (Grabbe et al., 2011; Zhang et al., 2014). While UCHL1 is known to be predominantly expressed in the brain and plays a central role in neurodegenerative diseases (Zhang et al., 2014; Das et al., 2006; Day and Thompson, 2010; Setsuie and Wada, 2007), studies have also established its function in ovarian development and fertility (Woodman et al., 2022). Recently, there has been an explosion of research elucidating the role of UCHL1 in cancer. Interestingly, the function of UCHL1 in cancer has been heavily debated as numerous studies, even within the same cancer subtype has shown that UCHL1 can operate both as a suppressor and promoter of tumorigenesis (Wang et al., 2023). Furthermore, UCHL1 represents a promising novel therapeutic approach, as it has the ability to be targeted with a selective small molecule inhibitor (Wang et al., 2023). While the efficacy of UCHL1 small molecule inhibition has been previously demonstrated in HGSOC (Tangri et al., 2021), there have collectively been a lack of studies that have evaluated this pharmacologic inhibition in heterogenous HGSOC models and the mechanistic adaptations that result from this blockade. Hence, in the current investigation, we sought to characterize the implications of UCHL1 small molecule inhibition in both chemosensitive and chemoresistant HGSOC cells, generating an unprecedented understanding of the clinically relevant implications of targeting UCHL1.

## Materials and methods

### Cell culture

HGSOC cell lines PEA1/2 were purchased from Millipore Sigma and cultured in RPMI1640 media supplemented with 2 mM of sodium pyruvate with 10% fetal bovine serum (FBS) and 1% penicillin/streptomycin. The OVCAR8 HGSOC cell line was obtained from American Type Culture Collection (ATCC) and cultured in Dulbecco Modified Eagle Medium (DMEM) supplemented with 10% FBS and 1% penicillin/streptomycin. All three cell lines were maintained in a 37°C/5% CO_2_ humidified chamber. Cells were treated with UCHL1 small molecule inhibitor at varying concentrations (5µM–40 µM as noted), LDN-57444 (Sigma Aldrich, L4170), 200 µM or 400 µM of carboplatin (Santa Cruz Biotechnology, CAS 4157.5-94-4) or DMSO control (Sigma Aldrich, D54879). All cell treatment timepoints were 48 h. Dose curve responses for each cell line to LDN-57444 can be seen in Supplementary Figure 1.

### S-Trap digestion and desalting

Samples (Treatment, labeled as “I” and Control labeled as “CD”, n = 4 per group) were transferred to the custody of the Proteomics Core Facility in approximately 100 μL o f Lysis Buffer (5% Sodium Dodecyl Sulfate (SDS, Invitrogen, 15553-035) in 50 mM Triethylammonium Bicarbonate Buffer pH 7.5 (TEAB, Sigma Aldrich, T7408-100 ML)) for proteomic profiling. Protein concentration was estimated using 2 μL of sample on a Nanodrop One (Thermo Fisher Scientific) and approximately 100 μg per sample was subject to enzymatic digestion via S-Trap (Protifi, C02-micro-40) as specified by the manufacturer with some modifications. Samples were reduced using 5 μL of Dithiothreitol (DTT, 10 mM final concentration, Sigma Aldrich, 646563-10X.5 ML) and incubated at 55°C for 45 min with mixing. Following incubation, samples were alkylated using 5uL of Iodoacetamide (IAM, 20 mM final concentration, Thermo Scientific, A39271) and incubated at room temperature in the dark for 30 min. Following alkylation, samples were acidified using 5 μL of Phosphoric Acid (2.5% final concentration, Sigma Aldrich, 79617-250 ML), and vortexed for 30 s 330 μL of ice-cold Binding Buffer (9 parts LC/MS Grade Methanol (ACROS Organics, 61513-0025):1 part 1M TEAB (100 mM Final concentration)) was added to each sample before the entire sample was transferred to each S-Trap spin column and centrifuged for 30 s at 4000xg. Each sample was then washed three times with 150 μL of binding buffer. Following protein washing, 1 vial (20 μg) of Trypsin (Promega, V5111) was reconstituted in 50 mM TEAB and added to each micro spin column to yield a final concentration of 1 μg of trypsin for 20 μg of protein in 100 μL. Samples were then moved to a humidified chamber in a 37*C incubator and left overnight (18 hr). On Day 2, samples were left to cool to room temperature for 15 min before 40 μL of 50 mM TEAB was added, and each sample was centrifuged for 1 min at 4000xg. Peptides were eluted with 40 μL of LC/MS grade Water (Honeywell, 39253-4X2.5L) and 0.1% Formic Acid (Fisher Scientific, A117-50), and centrifuged again for 1 min at 4000xg. Finally, to completely elute hydrophobic peptides, 40 μL of 50% LC/MS Grade Acetonitrile (Fisher, A955-4) was added to each sample and allowed to stand for 5 min before being centrifuged for 1 min at 4000×g. Each sample was concentrated using a speed vacuum concentrator (Thermo Fisher Scientific) for approximately 90 min. With solvent removed, samples were reconstituted in 100 μL of Solvent A (Water with 0.1% Formic Acid) and spiked with indexed retention time peptides (Biognosys, Ki-3002-1) in a 1:50 dilution to monitor HPLC performance.

### LC-MS proteomic analysis

Samples were then injected onto a QExactive Orbitrap LC/MS system (Thermo Fisher Scientific) and separated over a 120-min gradient consisting of solvent A (water + 0.1% Formic Acid), and solvent B (Acetonitrile and 0.1% Formic Acid) using a split-flow set-up on an Agilent 1200 series HPLC system. A 15 cm long (75um ID) capillary analytical column packed with XSelect CSH C18 2.5 μm resin (Waters) from the IDeA National Resource for Quantitative Proteomics at the University of Arkansas for Medical Sciences (UAMS) was used to separate digested peptides. The 120-min gradient consisted of 95% Solvent A for 1 min, 70% solvent A for 94 min, 5% solvent A for 6 min and finally 100% solvent A for the remaining 20 min all at a flow rate of 0.240 mL/min. The mass spectrometer acquisition utilized a Full MS/ddMS2 (Top9) centroid experiment. Full MS parameters used a default charge state of 2, 1 microscan, resolution of 70,000, AGC target of 3e6, 200 ms Maximum Injection time, in a scan range of 400–1800 m/z. ddMS2 parameters were 1 microscan, resolution of 17,500, AGC target of 2e4, a max injection time of 200 ms, a loop count of 9, Top 9 precursors, Isolation window of 2.5 m/z, collision energy of 28, and a scan range of 200–2000 m/z. Data dependent settings consisted of a minimum AGC target of 2e2, 1e3 intensity threshold, unassigned charge exclusion, all charge states, preferred peptide match, isotope exclusion, 30 s of dynamic exclusion.

### Label free quantitation and MaxQuant search parameters

Raw files were processed using MaxQuant (Ver 2.1.4) using 1% peptide and protein FDR search constraints, all values were default except for 20 ppm first search tolerance and 7 ppm main search tolerance against the latest version of the UniProt protein.fasta file for *Homo sapiens* (canonical and isoform, 20230316). Variable modifications consisted of just oxidation (M) and protein N-terminal Acetylation. Carbamidomethylation was the sole static modification for the Trypsin/P search. Match between runs algorithm was unselected and intensity-based absolute quantification (iBAQ) was selected. The proteingroups.txt file output was further processed in excel and Relative IBAQ was calculated as the intensity of each protein divided by the sum of all intensities for each given sample.

### Western blot

Protein extraction, Western blot, and imaging were performed as previously described (Ebott et al., 2024). Band density analysis was performed in ImageJ. All uncropped blots can be seen in Supplementary Material 1.

Antibodies and respective dilutions used were as follows:

NumbL- (Proteintech, 10111-1-AP, 1:500)
CEP55- (Proteintech, 23891-1-AP, 1:500)
ASNS- (Proteintech, 14681-1-AP, 1:500)
GPT2- (Proteintech, 16757-1-AP, 1:500)
GAPDH- (Santa Cruz Biotechnology, 47724, 1:1,000)
PSAT1- (Proteintech, 10501-1-AP, 1:500)
pSTAT3-(Cell Signaling, 9131S, 1:500)
STAT3-(Cell Signaling, 4904S, 1:500)
pAKT-(Proteintech, 28731-1-AP, 1:500)
AKT-(Cell Signaling, 9272S, 1:500)
pERK-(Proteintech, 28733-1-AP, 1:500)
ERK-(Proteintech, 11257-1-AP, 1:500)
Anti-Rabbit (Cell Signaling, 7074S, 1:1,000)
Anti-Mouse (Cell Signaling, 7076S, 1:1,000)

### Cell viability assays

HGSOC cell lines were seeded in a 96-well plate change to (20,000 cells/well). After 24-h cells were treated with a combination of LDN-57444 and carboplatin, or respective DMSO control. After 48 h of treatment, 10µL/well of CellTiter 96^®^ Aqueous One Solution proliferation MTS Assay (Promega, G3580) was added to the cells and incubated at 37°C/5% CO_2_ for 1 h and read at 490 nm in order to assess HGSOC cell viability. For 3D cell viability assays, cells were seeded in a 96 U-bottom plate (SBio, MS-9096UZ) (8,000 cells/well). After 24-h cells were treated with a combination of LDN-57444 and carboplatin, or respective DMSO control. PrestoBlue™ HS Viability Reagent Thermo Fisher Scientific, P50200) was added at a 1:100 concentration to the cells and incubated at 37°C/5% CO_2_ for 18 h and read at 570 nm and 600 nm and absorbance was normalized to the 600 nm wavelength in order to assess HGSOC cell viability 48 h after the combinatorial treatment.

### cBioPortal

cBioportal (Cerami et al., 2012; Gao et al., 2013) was employed to analyze the TCGA ovarian serous cystadenocarcinoma cohort from the Nature 2011 (n = 489) study in order to determine UCHL1’s association with platinum status.

### KEGG analysis

KEGG analysis was performed in Database for Annotation, Visualization, and Integrated Discovery (DAVID; https://davidbioinformatics.nih.gov/) (Sherman et al., 2022; Huang et al., 2009) All peptides with significantly differential expression (p < 0.05) were analyzed as a “gene list” for the DAVID “Functional Annotation Tool.” Further analysis was performed via DAVID’s “KEGG_PATHWAY” function to produce a list of significantly enriched KEGG pathways (p < 0.05).

### Statistical analysis

GraphPad Prism was employed for all statistical analyses. Student t-tests were performed to determine differences in percent abundance rates for proteomic analysis and relative band density for Western blot replicates. All p-values reported were 2-tailed and unadjusted. KEGG analysis reports adjusted p-values using the Benjamini-Hochberg test with count = 2 and ease = 0.1.

## Results

### Proteomic implications of small molecule UCHL1 inhibition in HGSOC cells

**FIGURE 1:**
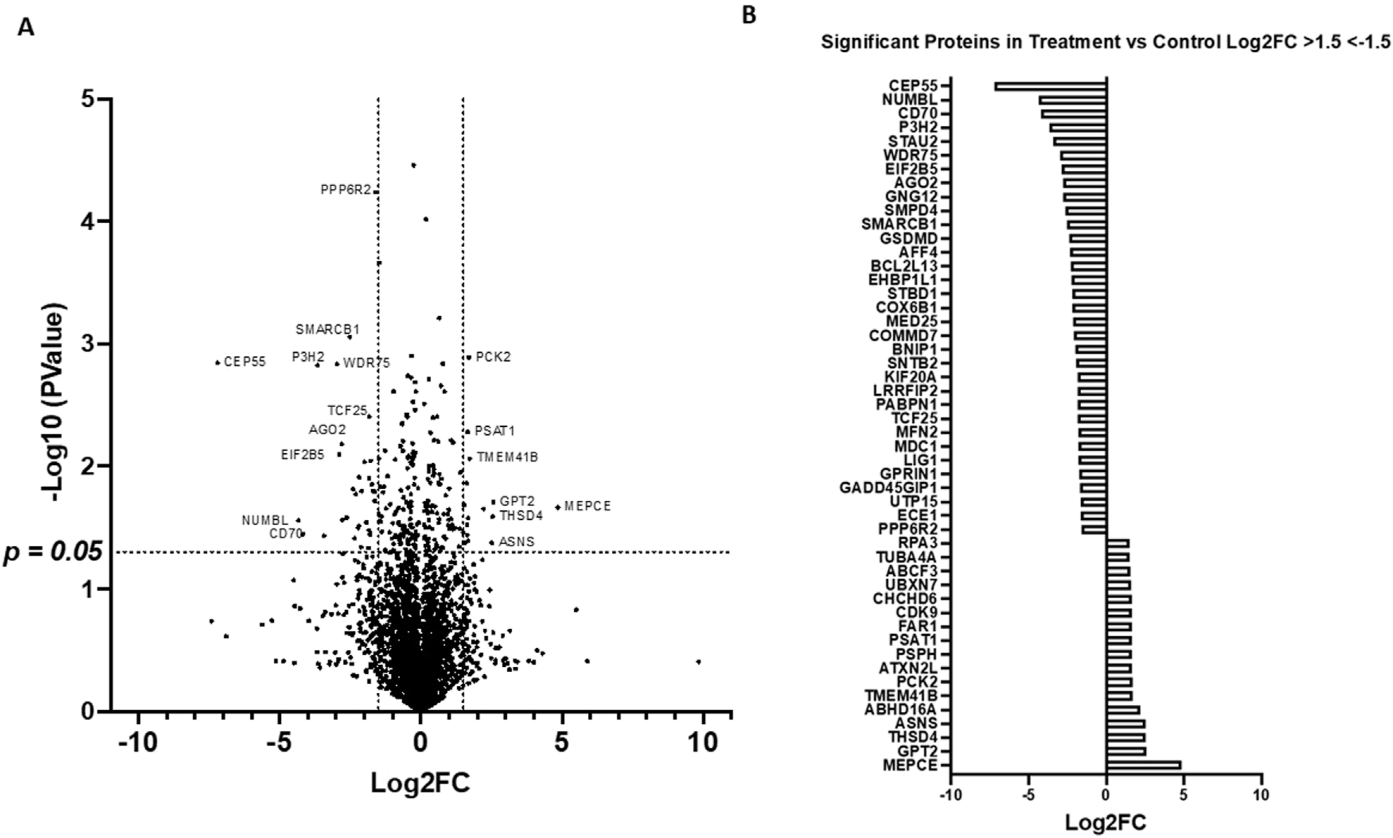
LC-MC proteomic analysis of OVCAR8 subjected to small molecule UCHL1 inhibition. **(A)** Volcano plot demonstrating differential protein expression in OVCAR8 cells treated with 10 µM of LDN-57444 relative to DMSO control for 48 h, measured by LC-MS. **(B)** Top differentially expressed proteins in OVCAR8 cells treated with LDN-57444 relative to DMSO control. LC, liquid chromatography, MS, mass spectrometry.

**FIGURE 2:**
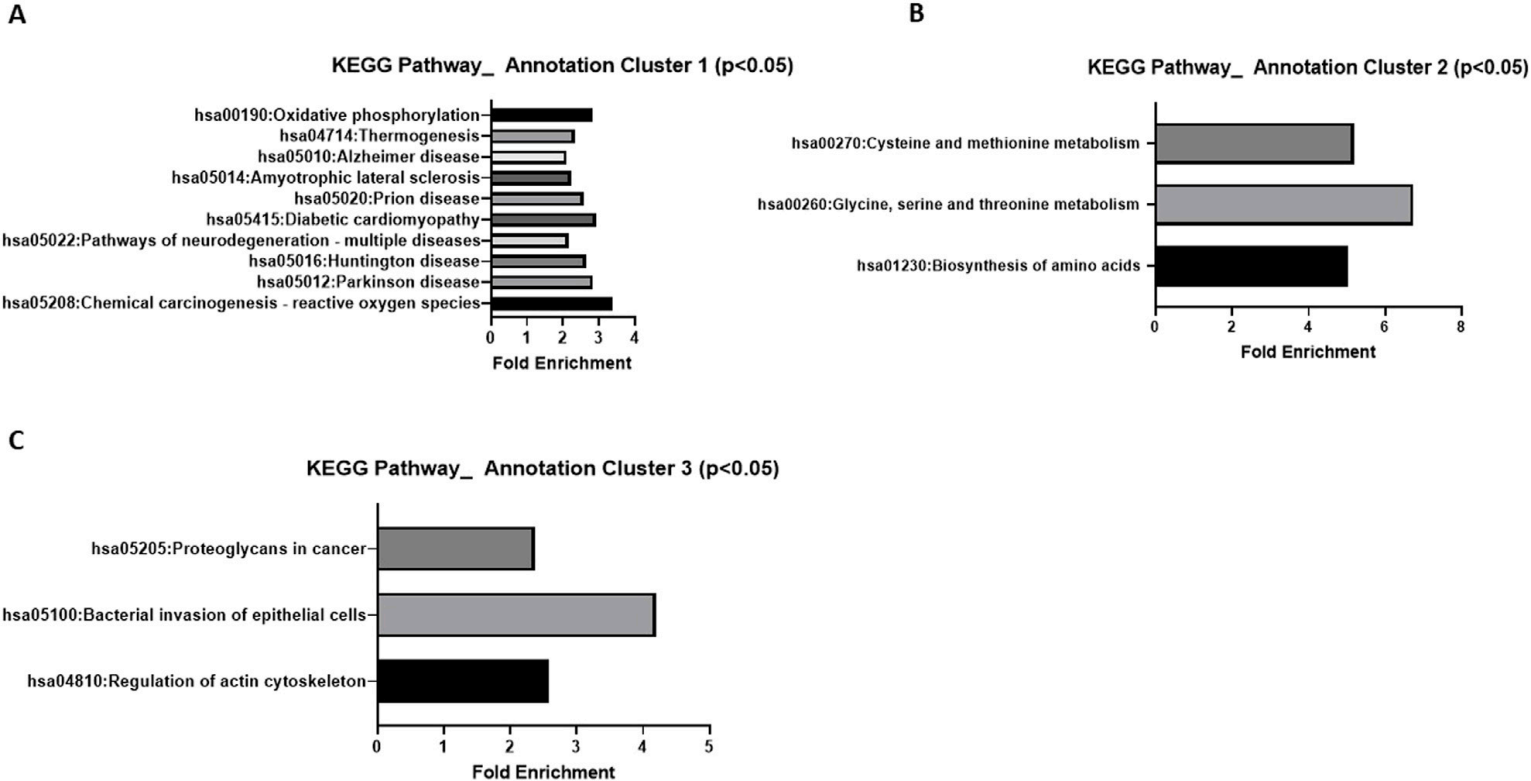
KEGG analysis of LC-MC proteomic results. Significant (p < 0.05) pathway fold enrichment in annotation cluster **(A)** 1 **(B)** 2, and **(C)** 3 of differentially expressed proteins in OVCAR8 cells treated with LDN-57444 relative to DMSO control.

A comparative label-free proteomic analysis was performed in HGSOC OVCAR8 cells treated with 10 µM of LDN-57444, which revealed significant (p < 0.05) log2 fold-change (FC) in protein expression, such as in, centrosomal protein of 55 kda (CEP55, −7.21 FC), CD70 antigen (CD70,−4.18 FC), numb-like protein (NumbL, −4.34 FC), phosphoserine aminotransferase (PSAT1, 1.66 FC), alanine aminotransferase 2/glutamic-pyruvic transaminase 2 (GPT2, 2.57 FC), asparagine synthetase (ASNS, 2.51 FC), and methyl phosphate capping enzyme (MEPCE, 4.86 FC) (Figure 1). A comprehensive list of all protein changes can be seen in Supplementary Material 2. Interestingly, PSAT1, GPT2, and ASNS are all prominent proteins involved in cell metabolism (Zhang et al., 2020; Cao et al., 2017; Chi et al., 2020), while NumbL and CEP55 are implicated in cell division (García-Heredia et al., 2016), and cell cycle progression (Zhang et al., 2023), respectively. MEPCE is a methyltransferase that adds a monomethyl cap to small nuclear RNA (Patel et al., 2023), and CD70 is expressed readily on tumor cells and enhances cancer and regulatory T cell survival (Jacobs et al., 2015). KEGG analysis revealed that one annotation cluster exhibited significant (p < 0.05) fold enrichment in pathways related to oxidative phosphorylation, thermogenesis, neurodegeneration-multiple diseases, Huntington disease, Parkinson disease, and Alzheimer disease (Figure 2A). The analysis added validity and served as a “positive control” to our results, as it is well known that UCHL1 is implicated in neurodegenerative diseases (Liu et al., 2002; Zhang et al., 2014; Das et al., 2006; Day and Thompson, 2010; Setsuie and Wada, 2007). In addition, annotation cluster 2 revealed further significant (p < 0.05) changes in cellular metabolic pathways such as cysteine and methionine metabolism, glycine, serine, and threonine metabolism, and biosynthesis of amino acids (Figure 2B). Finally in the third annotation cluster significant (p < 0.05) fold enrichment was found in pathways related to proteoglycans in cancer, bacterial invasion of epithelial cells, and regulation of actin cytoskeleton (Figure 2C). Complete KEGG analysis output can be seen in Supplementary Material 3.

**FIGURE 3:**
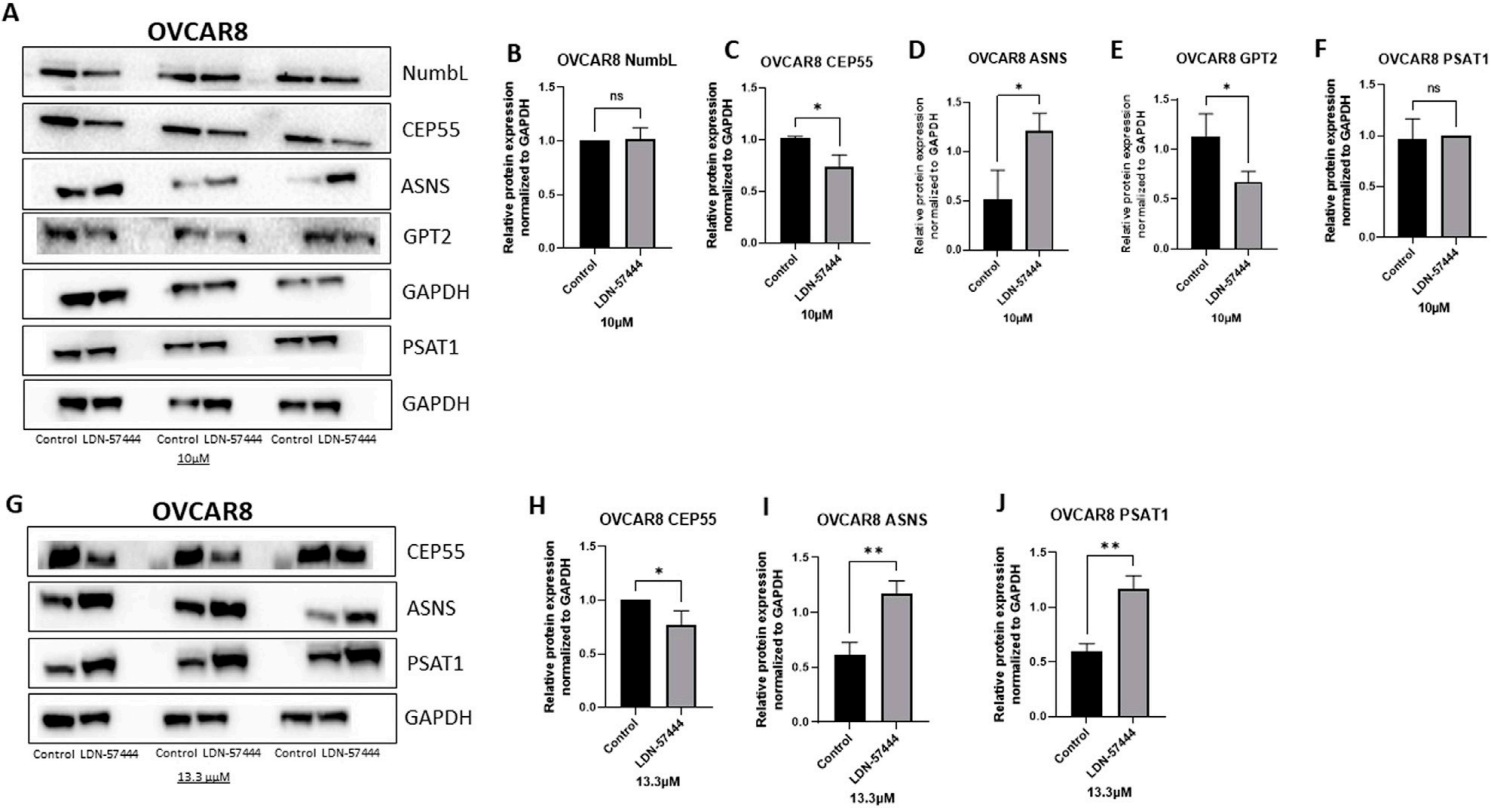
Validation of enriched proteins identified by proteomic analysis in OVCAR8 cells. Western blot analysis of **(A)** NumbL, CEP55, ASNS, GPT2, and PSAT1, with respective GAPDH loading controls in OVCAR8 cells treated with either 10 µM of LDN-57444 or DMSO control for 48 h. Relative band densities of **(B)** NumbL, **(C)** CEP55, **(D)** ASNS, **(E)** GPT2, and **(F)** PSAT1, normalized to GAPDH. Western blot analysis of **(G)** CEP55, ASNS, and PSAT1, with respective GAPDH loading controls in OVCAR8 cells treated with either 13.3 µM of LDN-57444 or DMSO control. Relative band densities of **(H)** CEP55, **(I)** ASNS, and **(J)** PSAT1, normalized to GAPDH. Error bars represent standard deviation of 3 biological replicated. *p < 0.05, **p < 0.005, as indicated. ns, non-significant.

Next, Western blot analysis was employed to validate proteomics results in OVCAR8 cells at 10µM and 13.3 µM concentrations of LDN-57444 treatment. Following 10 uM LDN-57444 it was determined that there was a significant (p < 0.05) decrease in CEP55 expression, and a significant (p < 0.05) increase in ASNS and GPT3, compared to the DMSO control, with no significant changes in NumbL and PSAT1 detected (Figures 3A–F). However, upon treatment with an elevated LDN-57444 concentration of 13.3 µM, a similar significant (p < 0.05) decrease in CEP55 expression was observed, with a corresponding striking significant (p < 0.005) upregulation of ASNS and PSAT1 in treated cells compared to DMSO control (Figures 3G–J).

**FIGURE 4:**
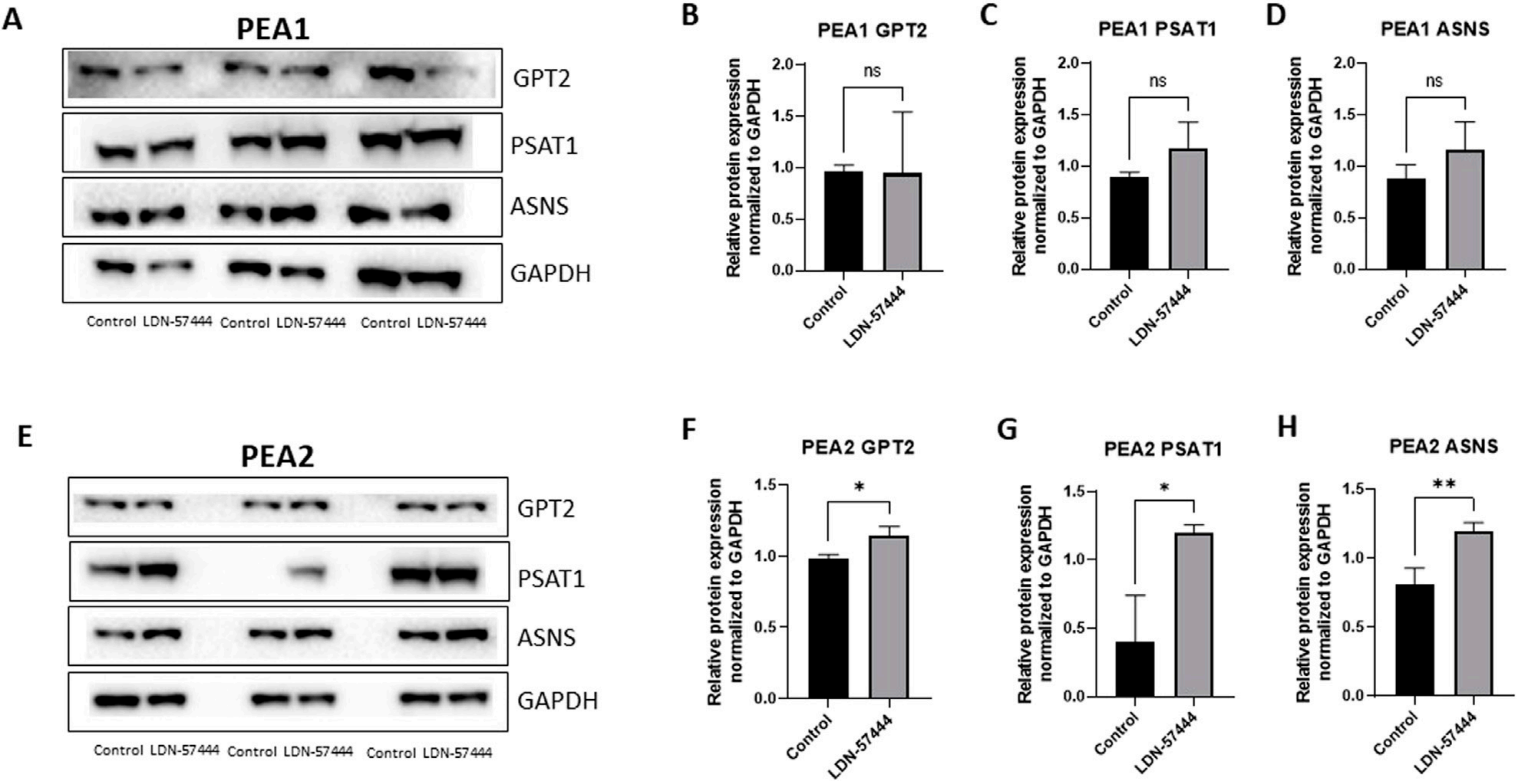
Validation of enriched proteins identified by proteomic analysis in PEA1 and PEA2 cells. Western blot analysis of **(A)** GPT2, PSAT1, and ASNS with respective GAPDH loading controls in PEA1 cells treated with either 10 µM of LDN-57444 or DMSO control for 48 h. Relative band densities of **(B)** GPT2, **(C)** PSAT1, and **(D)** ASNS normalized to GAPDH. Western blot analysis of **(E)** GPT2, PSAT1, and ASNS with respective GAPDH loading controls in PEA2 cells treated with either 5 µM of LDN-57444 or DMSO control. Relative band densities of **(F)** GPT2, **(G)** PSAT1, and **(H)** ASNS normalized to GAPDH. Error bars represent standard deviation of 3 biological replicated. *p < 0.05, **p < 0.005, as indicated. ns, non-significant.

In addition to validating our proteomic findings in OVCAR8 cells, we also sought to determine how these cellular metabolism proteins change in other HGSOC cells, matched chemosensitive and chemoresistant clones, PEA1, and PEA2, respectively, in which PEA2 cells were obtained at the time of progression from platinum-based therapy at 6 months. Therefore, these matched cell lines allowed for the investigation of UCHL1 small molecule inhibition as HGSOC progresses from a chemosensitive to resistant state (Cooke et al., 2010) Interestingly, in the PEA1 chemosensitive HGSOC cells there were no significant difference in GPT2, PSAT1, or ASNS levels following LDN-57444 treatment compared to DMSO control (Figures 4A–D), while conversely, in the PEA2 chemoresistant cells GPT2, PSAT1, and ASNS, were all significantly (p < 0.05) upregulated in response to UCHL1 small molecule inhibition, matching our OVCAR8 results (Figures 4E–H). The concordance between our OVCAR8 and PEA2 cells can partially be explained by the fact that OVCAR8 cells are commonly thought to be moderately chemoresistant, as they were obtained from a HGSOC patient after a high dose of carboplatin (Schilder et al., 1990; Nunes et al., 2021). Taken together, these proteomic results suggest that pharmacologic inhibition of UCHL1 leads to pronounced implications in cell metabolism in HGSOC, particularly in tumor cells that possess a chemoresistant phenotype.

### Combinatorial UCHL1 inhibition and carboplatin demonstrates differential effects on HGSOC cell death according to platinum status

**FIGURE 5:**
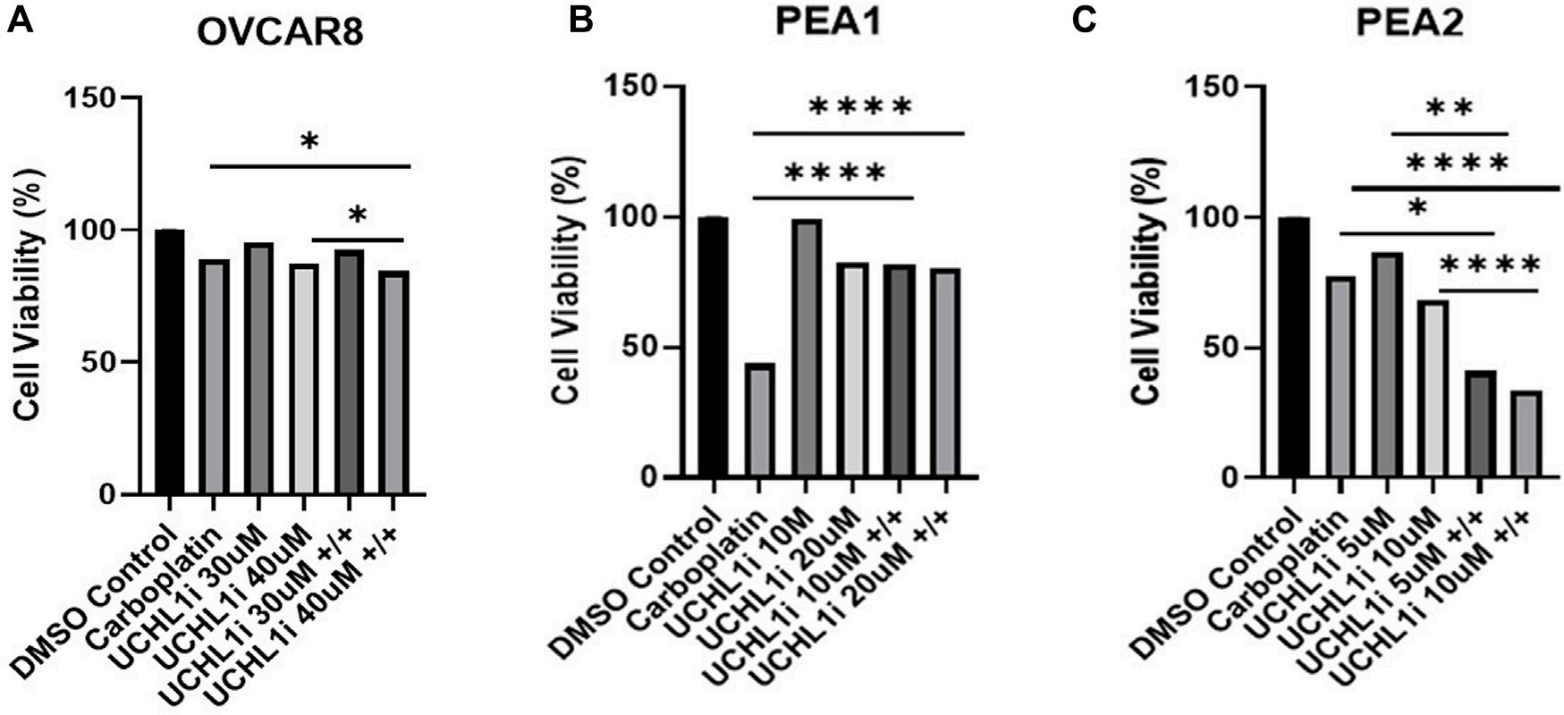
Combinatorial efficacy of dual LDN-57444 inhibition and carboplatin in HGSOC cell lines. Cell viability analysis of **(A)** OVCAR8 **(B)** PEA1, and **(C)** PEA2 cells treated with carboplatin (200 µM for OVCAR8/PEA1, 400 µM for PEA2) and varying LDN-57444 concentrations as noted alone and in combination, with respective DMSO control for 48 h. Error bars represent standard deviation of ≥3 biological replicates. *p < 0.05, **p < 0.005, *****p < 0.00005, as indicated.

Next, we sought to assess tumor cell viability following UCHL1 small-molecule inhibition and carboplatin treatment in HGSOC cell lines. Interestingly, in OVCAR8 cells we observed a significant (p < 0.05), albeit non-striking 4.8% decrease in LDN-57444 (40 µM) and carboplatin treatment compared to carboplatin alone, and a 2.9% decrease in cell viability in dual LDN-57444 (40 µM) and carboplatin treatment compared to LDN-57444 (40 µM) treatment alone (Figure 5A). Conversely, in the PEA1 cells we observed a rescuing effect of UCHL1 inhibition on cell survival, as dual LDN-57444 at the 10 µM and 20 µM concentration with carboplatin lead to a significant (p < 0.0001) 38.2%, and 36.6% increase in viability, respectively, compared to carboplatin treatment alone (Figure 5B). Finally, in chemoresistant PEA2 cells, treatment with a 10 µM concentration of LDN-57444 and carboplatin led to a significant (p < 0.0001) 58.8% and 69.1% decrease in cell viability compared to carboplatin alone and LDN-57444 treatment alone, respectively (Figure 5C). In addition, we observed similar trends following treatment with both LDN-57444 and carboplatin in OVCAR8, PEA1, and PEA2 spheroids (Supplementary Figure 2). Overall, these results revealed differential effects on HGSOC cell viability upon combinatorial UCHL1 inhibition and chemotherapy according to platinum sensitivity.

### Small molecule inhibition produces differential expression in activated cell growth pathways in chemosensitive versus chemoresistant HGSOC lines

**FIGURE 6:**
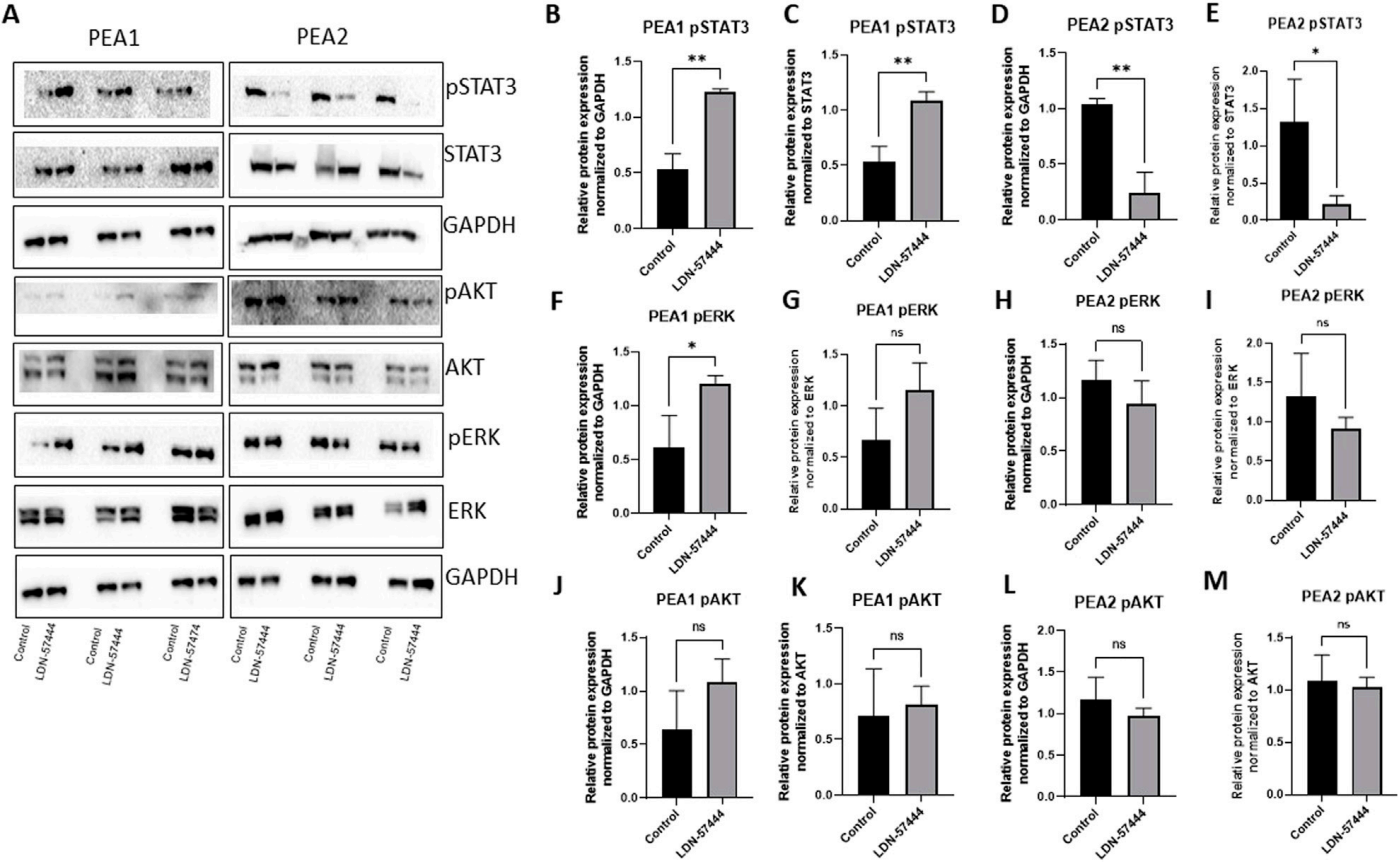
Cell growth pathway changes in PEA1 and PEA2 in response to UCHL1 small molecule inhibition **(A)** pSTAT3/STAT3, pAKT/AKT, and pERK/ERK with respective GAPDH loading controls in PEA1 and PEA2 cells treated with LDN-57444 (10 µ PEA1, 5 µM, PEA2) or DMSO control. Relative band densities of pSTAT3 in PEA1 normalized to **(B)** GAPDH and **(C)** total STAT3. PEA2 pSTAT3 levels normalized to **(D)** GAPDH and **(E)** total STAT3. PEA1 pERK levels normalized to **(F)** GAPDH and **(G)** total ERK. PEA2 pERK levels normalized to **(H)** GAPDH and **(I)** total ERK. PEA1 pAKT levels normalized to **(J)** GAPDH and **(K)** total ERK. PEA2 pAKT levels normalized to **(L)** GAPDH and **(M)** total AKT. Error bars represent standard deviation of 3 biological replicated. *p < 0.05, **p < 0.005, as indicated. ns, non-significant.

**FIGURE 7:**
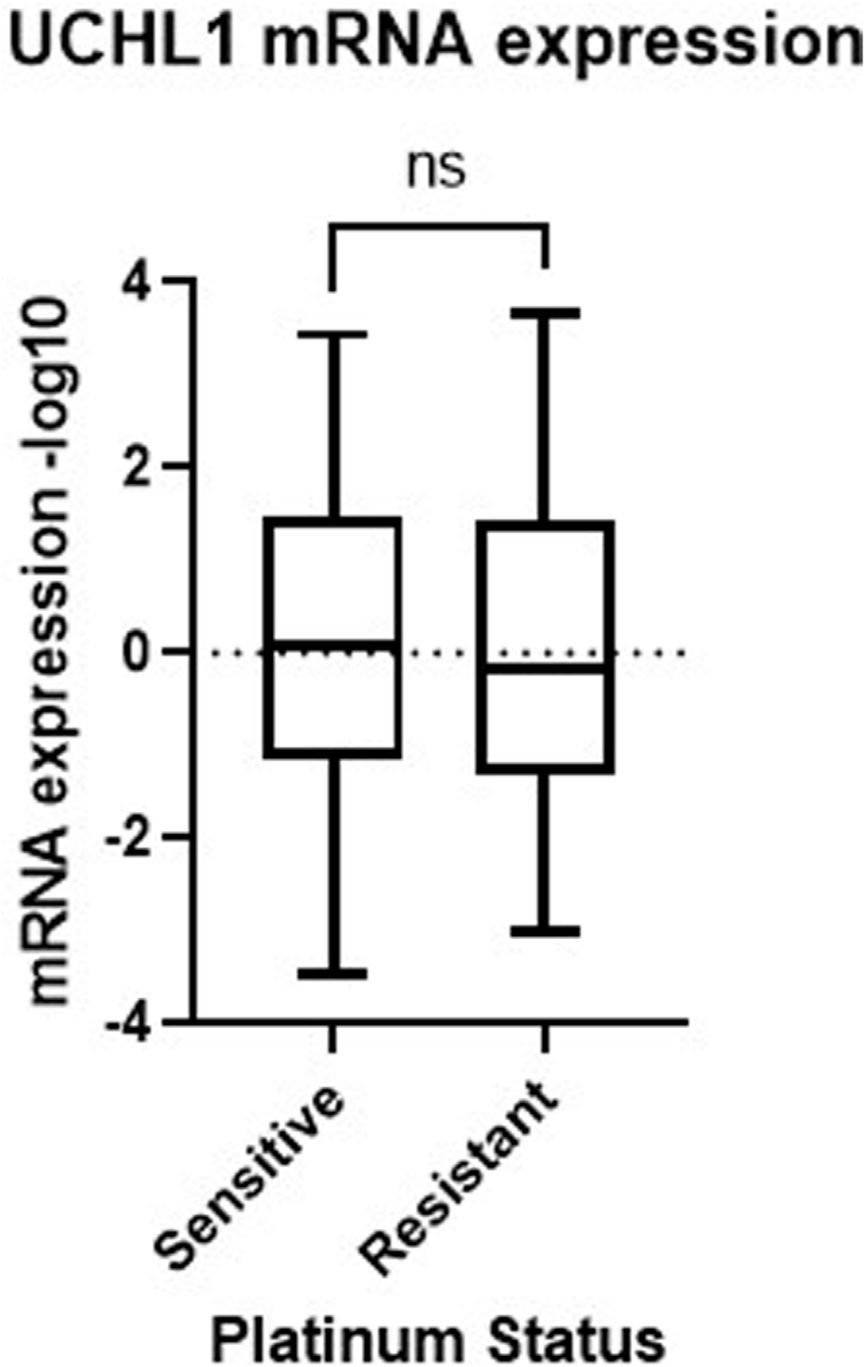
*UCHL1* expression in serous ovarian cancer patient tumors. *UCHL1* mRNA expression (−log10) in patient platinum sensitive (n = 197) and resistant (n = 90) tumors obtained from the TCGA Nature 2011 cohort. TCGA, The Cancer Genome Atlas; ns, non-significant.

To follow up on our cell viability results, we performed Western blot analysis to observe how common tumor cell growth pathways are affected by UCHL1 small-molecule inhibition in both chemosensitive (PEA1) and chemoresistant (PEA2) cells. Fascinatingly, in PEA1 cells we observed significant (p < 0.05) increases in phospho(p)STAT3 following LDN-57444 treatment compared to DMSO control, as well as an increase in pAKT expression, which was non-significant. While pERK levels were significantly (p < 0.05) increased following LDN-57444 treatment upon normalization to GAPDH levels, this increase was non-significant when normalized to total ERK. Conversely, upon LDN-57444 treatment in PEA2 cells we observed a significant (p < 0.05) decrease in pSTAT3 expression as well as a decrease in pERK and pAKT that was non-significant (Figure 6). These findings corroborated our cell viability analyses and suggested that in chemosensitive HGSOC cells that UCHL1 inhibition promotes tumor cell growth, while in chemoresistant HGSOC it combats tumorigenesis. Importantly, we did not observe differences in UCHL1 expression in PEA1 and PEA2 cells (Supplementary Figure 3), and upon bioinformatic analysis of the TCGA ovarian serous cohort, we did not observe significant differences in *UCHL1* expression in chemosensitive versus chemoresistant tumors (Figure 7), suggesting that UCHL1 expression is not responsible for the differential effects observed in chemosensitive versus chemoresistant HGSOC following exposure to UCHL1 pharmacologic inhibition.

## Discussion

This study is the first to report that the efficacy of UCHL1 small molecule inhibition in HGSOC is dependent upon platinum status. Despite this finding, it has been well documented that UCHL1 possesses dual functions as either an oncogene or tumor suppressor. In colorectal cancer (CRC) (Zhong et al., 2012), non-small cell lung cancer (NSCLC) (Kim et al., 2008), neuroendocrine carcinoma (Liu et al., 2024), lymphoma (Hussain et al., 2010), gastric cancer (Gu et al., 2015), and ER+ and triple-negative breast cancer (Mondal et al., 2021), UCHL1 has been reported to function as an oncogene, while in contrast, in nasopharyngeal (Li et al., 2010), hepatocellular (Yu et al., 2008), and prostate (Ummanni et al., 2011) cancers, it has been described as a tumor suppressor. Interestingly, numerous studies have concluded that UCHL1’s deubiquitinase activity is responsible for its promotion of oncogenesis (Zhong et al., 2012; Hussain et al., 2010; Gu et al., 2015). Specifically, in gastric cancer, it has been revealed that overexpression of UCHL1 increases cell proliferation, migration, and invasion by activating the AKT and ERK1/2 tumor growth pathways, a phenomenon dependent on the enzymatic activity of UCHL1 (Gu et al., 2015). Furthermore, Hussain et al. found that UCHL1 supports lymphoma development through AKT activation and downregulation of the phosphatase PHLPP1, which also requires deubiquitinase activity (Hussain et al., 2010). Finally, Zhong et al. determined that UCHL1’s deubiquitinase activity was responsible for CRC pathogenesis through activation of the β-catenin-TCF pathway (Gu et al., 2015). In this current investigation, we observed a decrease in activated tumor growth pathways in chemoresistant PEA2 HGSOC cells following UCHL1 small-molecule inhibition. Therefore, future studies should determine whether UCHL1’s oncogenic function in chemoresistant HGSOC cells is mechanistically driven by its deubiquitinase activity. Conversely, prior studies have speculated that UCHL1 functions as a tumor suppressor, as it is has been demonstrated that UCHL1 forms a complex with and stabilizes p53 (Li et al., 2010; Xiang et al., 2012). However, as all three HGSOC cell lines used in this study harbored p53 mutations (Cooke et al., 2010; Leroy et al., 2015) this theory is unlikely to explain the differences in cell growth and viability observed in this study following UCHL1 small molecule inhibition.

The role of UCHL1, as specifically implicated in ovarian pathogenesis, is similarly complex and multifaceted, as demonstrated by this current study. Furthermore, a study by Jin et al. in non-HGSOC epithelial ovarian cancer cell lines found that the knockdown of UCHL1 halted tumor cell apoptosis and an increase in cisplatin resistance (Jin et al., 2013). In addition, the authors observed that UCHL1 expression in various tested cell lines was inversely correlated with their cisplatin-resistant levels. While we did not find any meaningful differences in expression in our cell lines used, this discordance could be potentially be explained by the fact that the majority of cell lines used in Jin et al. were of non-serous histology. On the contrary, a study by Tangri et al. in HGSOC revealed that UCHL1 is upregulated in HGSOC tumors and contributes to ovarian pathogenesis through its mediation of protein homeostasis via the PSMA7-APEH axis (Tangri et al., 2021). In addition, it was found that using an *in vivo* OVCAR8 xenograft model, LDN-57444 combated HGSOC tumor growth and increased apoptosis rates (Tangri et al., 2021). Finally, as the authors reported both UCHL1 promoter hypomethylation and the epigenetic upregulation of UCHL1, they theorized that mutant p53 could be responsible for the transcriptional induction of UCHL1 in HGSOC (Tangri et al., 2021). Nevertheless, this proposed mutant p53 induction of UCHL1 fails to explain the differences we observe in UCHL1 pharmacologic inhibition in chemosensitive versus chemoresistant HGSOC cells, underscoring the importance of future mechanistic studies to be initiated to unravel this phenomenon.

While to the best of our knowledge, we are the first to report efficacy of small molecule UCHL1 inhibition specifically in chemoresistant HGSOC cells, there have been a number of studies in cancer that have described UCHL1’s complex role in both chemo- and drug-resistance (Ding et al., 2020; Lu et al., 2021; Yang et al., 2018; Lee et al., 2021; Cucci et al., 2020; Ning et al., 2017; Jin et al., 2015; Brinkmann et al., 2013). A study by Ning et al. reported that breast cancer adriamycin-resistant cells secreted UCHL1 containing exosomes that have the ability to be become incorporated into adriamycin-sensitive breast cancer cells within the tumor microenvironment (Ning et al., 2017). Conversely, in hepatocellular carcinoma it was found that overexpression of UCHL1 combated chemoresistance to verapamil and adriamycin and enhanced apoptosis rates (Yang et al., 2018). In addition to the differential effects of small molecule UCHL1 inhibition in chemosensitve versus chemoresistant HGSOC cells that our group observed, this investigation also revealed that prominent intratumoral changes in cellular metabolism proteins was a consequence of a UCHL1 blockade in cells specifically with a chemoresistant phenotype. Intriguingly, a number of studies that have described the role of UCHL1 and chemoresistance, have noted that this association is tied together by metabolic adaptations. Specifically, in NSCLC it was found that UCHL1 promoted pemetrexed-based chemotherapy resistance via the upregulation of thymidylate synthase (Ding et al., 2020) and in breast cancer drove doxorubicin resistance through the stimulation of free-fatty acid production (Lu et al., 2021). Finally, a study by Nakashima et al. identified that UCHL1 is a novel upstream activator of hypoxia-inducible factor 1 and demonstrated that UCHL1 overexpression led to carbohydrate metabolism reprogramming and an increase in NADPH levels dependent upon the pentose phosphate pathway (PPP) (Nakashima et al., 2017). Finally, the group demonstrated in breast cancer cells that inhibition of the PPP perturbed UCHL1 mediated radioresistance (Nakashima et al., 2017). Taken together, these studies suggest an association exists between cellular metabolism changes driven by UCHL1 and chemo or drug resistance. While a limitation of this current study is that the mechanistic link between UCHL1 inhibition and the changes in prominent cell metabolism pathways in both HGSOC chemosensitive and chemoresistant preclinical models was not investigated, this will be an imperative future direction.

One additional limitation of this current study is that we did not compare small molecule inhibition with that of a UCHL1 knockdown, which will be important to include in future directions of this research in order to elucidate the role of UCHL1 in HGSOC chemoresistance. However, for the purposes of this study we chose to focus on small molecule inhibition of UCHL1 as it is most clinically applicable and translatable to HGSOC patient care. Furthermore, the differential effects observed in chemosensitve and chemoresistant HGSOC cells *in vitro* upon combinatorial chemotherapy and UCHL1 blockade will need to be explored *in vivo* in order to uncover any potential off target effects at prolonged exposure timepoints. Taken together, this study demonstrates the anti-tumor efficacy of small molecule UCHL1 in HGSOC, specifically in the context of chemoresistance. While a key future direction stemming from this study involves elucidating the mechanism behind differences in efficacy of LDN-57444 in chemosensitive versus chemoresistant HGSOC cells, this investigation suggests that UCHL1 is a novel therapeutic target to combat HGSOC chemoresistance, an area of urgent clinical need.

## Data Availability

The data presented in the study are deposited in the ProteomeXchange Consortium via the PRIDE partner repository, accession number PXD060691.

## Supplementary material

SUPPLEMENTARY FIGURE 1 Dose response curves of LDN-57444 in HGSOC cell lines. Cell viability analysis of **(A)** PEA1 **(B)** PEA2, and **(C)** OVCAR8, in response to varying doses of LDN-57444 and DMSO control.

SUPPLEMENTARY FIGURE 2 Combinatorial efficacy of dual LDN-57444 inhibition and carboplatin in HGSOC cell lines. Cell viability analysis of **(A)** OVCAR8 **(B)** PEA1, and **(C)** PEA2 spheroids treated with carboplatin (200 µM for OVCAR8/PEA1, 400 µM for PEA2) and varying LDN-57444 concentrations as noted alone and in combination, with respective DMSO control for 48 h. Error bars represent standard deviation of ≥3 biological replicates. *p < 0.05, **p < 0.005, ***p < 0.0005, ****p < 0.00005, as indicated.

SUPPLEMENTARY FIGURE 3 Basal expression of UCHL1 in HGSOC cell lines. Western blot analysis of UCHL1 in PEA1, PEA2, and OVCAR8 cells, with respective GAPDH loading controls.
