# Supplementary figures and images for: Small molecule inhibition of ubiquitin C-terminal hydrolase L1 alters cell metabolism proteins and exerts anti- or pro-tumorigenic effects contingent upon chemosensitivity status in high grade serous ovarian cancer
Source: Front Pharmacol. 2025 Feb 26;16:1547164. doi: 10.3389/fphar.2025.1547164 (PMC11897294; doi:10.3389/fphar.2025.1547164)

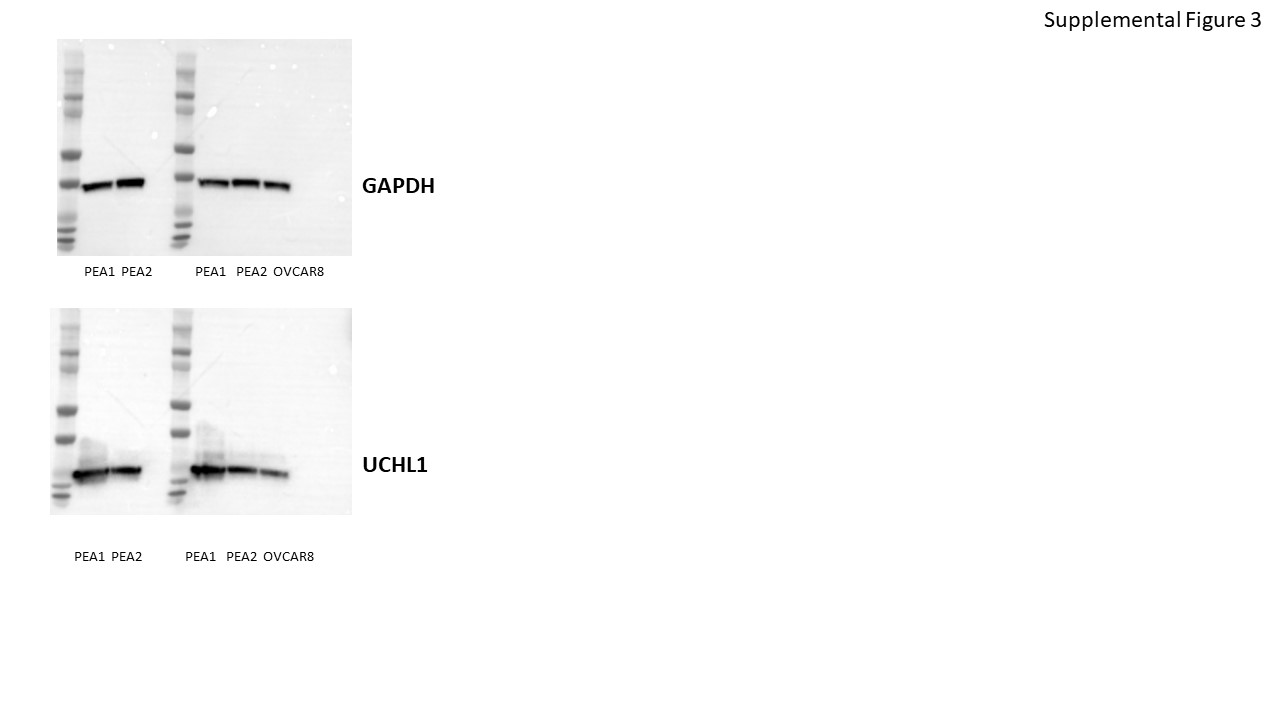

Supplement: Supplementary file 1 [file Image3.jpeg]

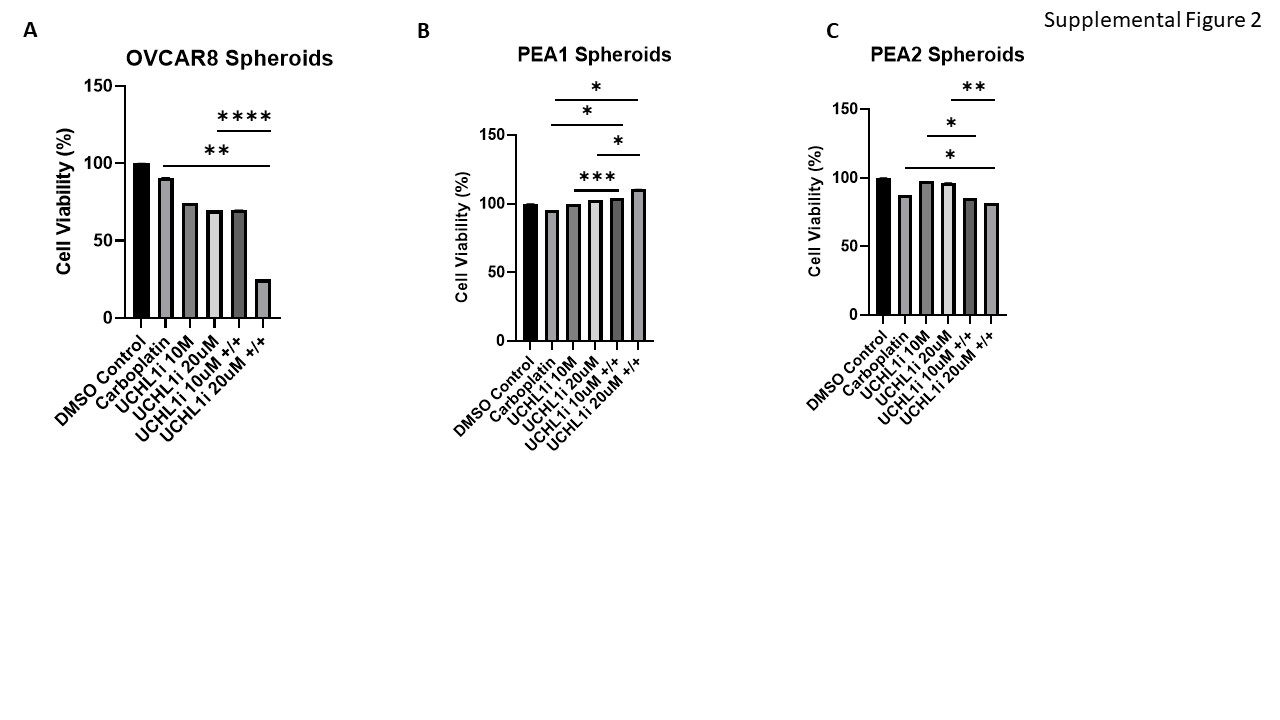

Supplement: Supplementary file 2 [file Image2.jpeg]

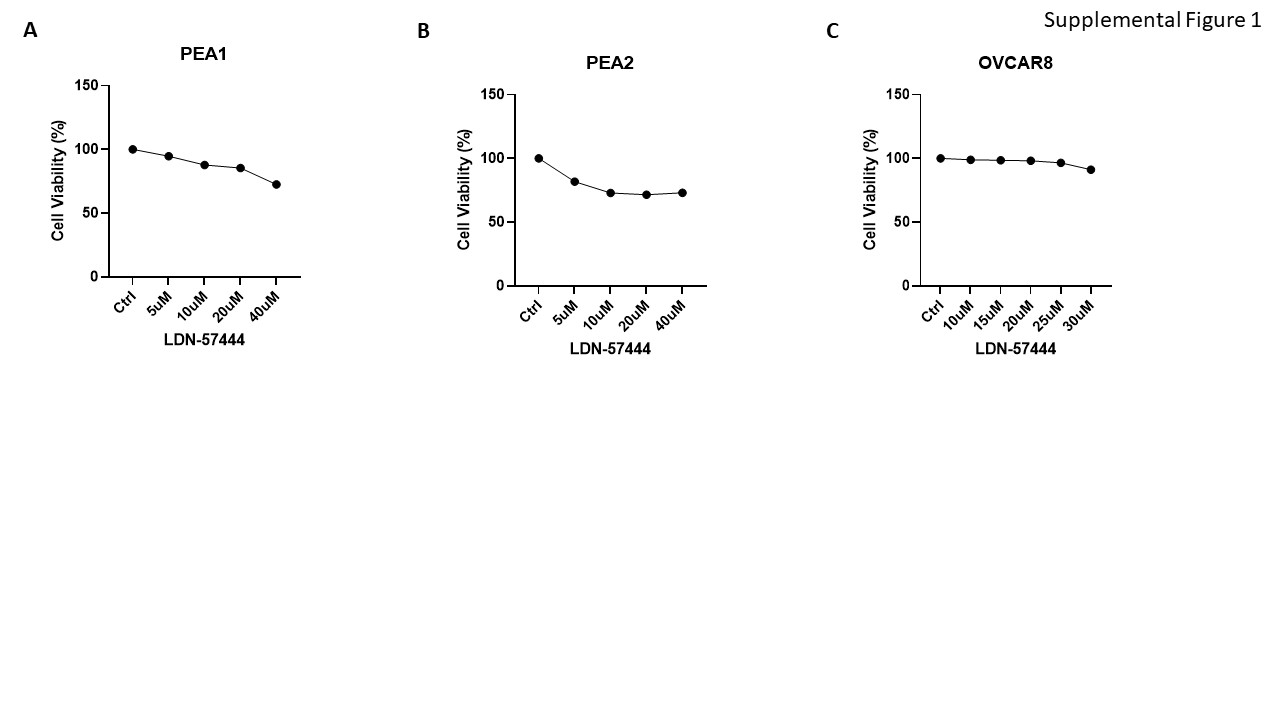

Supplement: Supplementary file 5 [file Image1.jpg]
