# Supplementary material for: Small molecule inhibition of ubiquitin C-terminal hydrolase L1 alters cell metabolism proteins and exerts anti- or pro-tumorigenic effects contingent upon chemosensitivity status in high grade serous ovarian cancer
Source: Front Pharmacol. 2025 Feb 26;16:1547164. doi: 10.3389/fphar.2025.1547164 (PMC11897294; doi:10.3389/fphar.2025.1547164)
Supplement: Supplementary file 4 [file DataSheet1.pdf]

PEA2

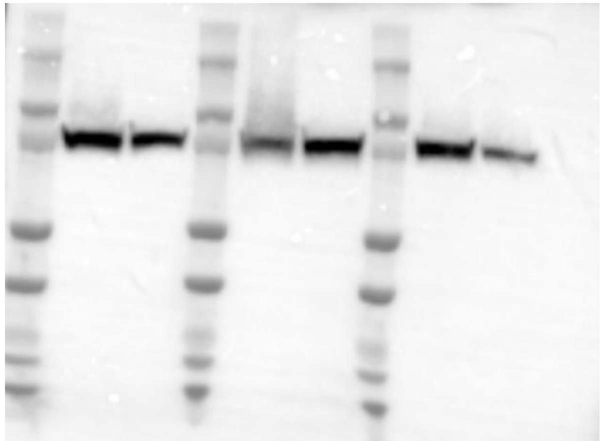

STAT3

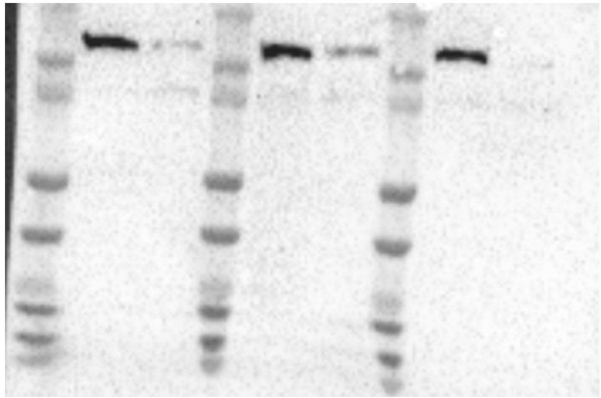

pSTAT3

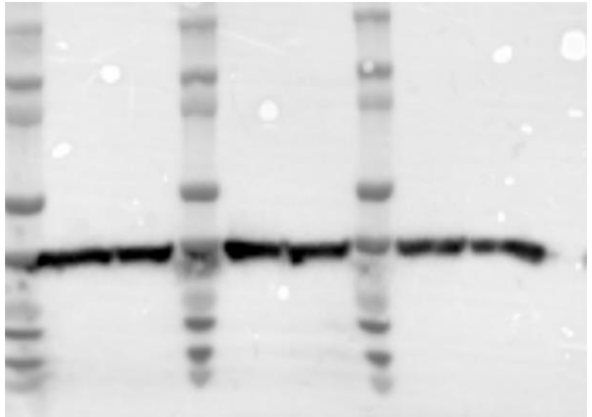

GAPDH

PEA2

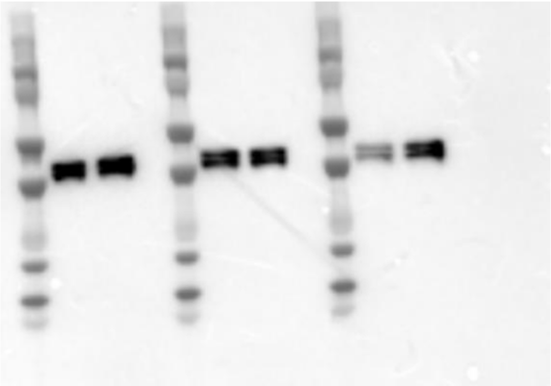

ERK

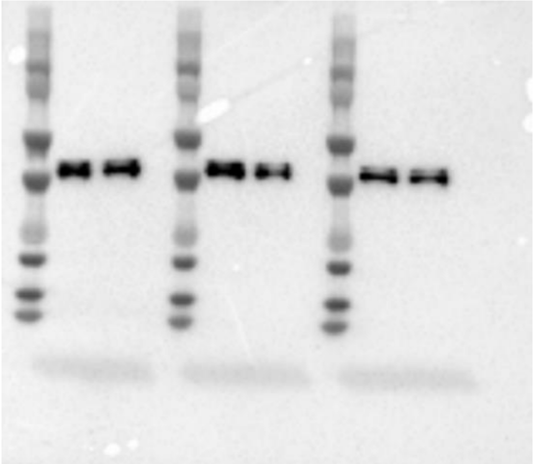

pERK

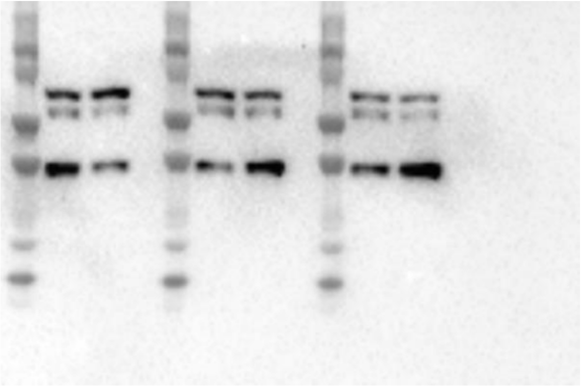

AKT

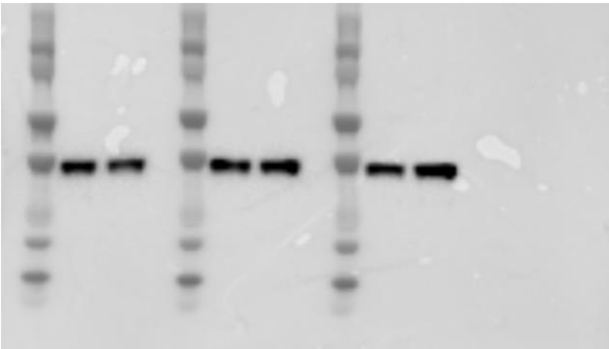

GAPDH

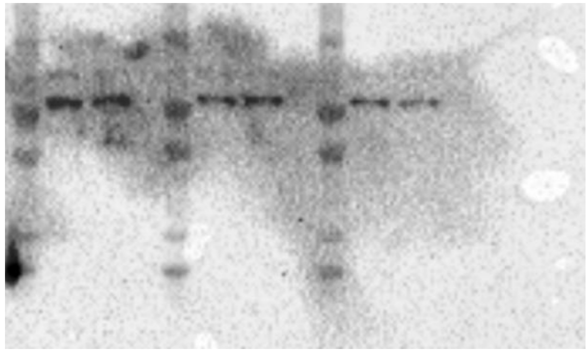

pAKT

PEA1

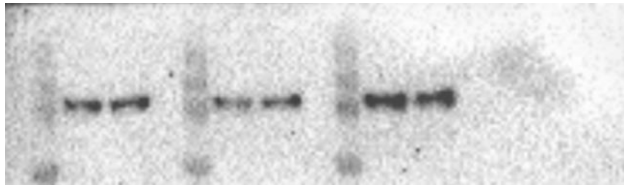

STAT3

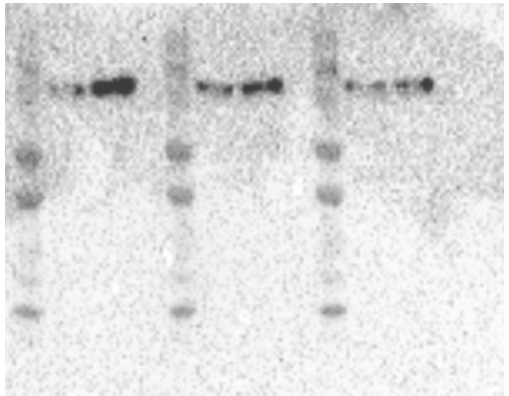

pSTAT3

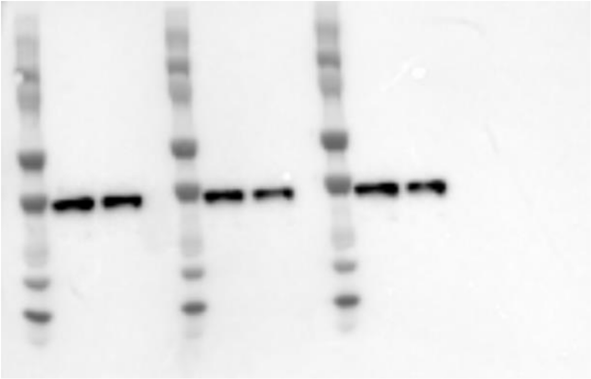

GAPDH

PEA1

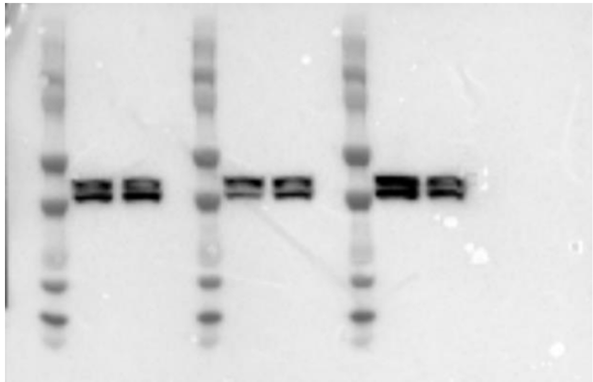

ERK

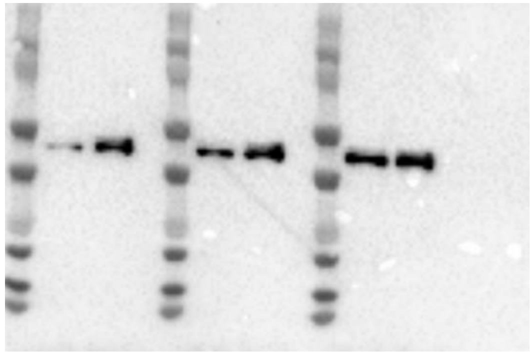

pERK

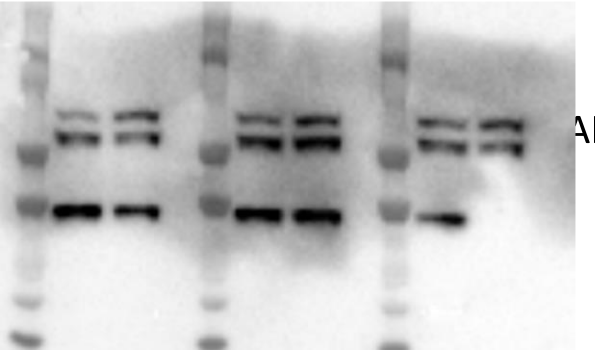

AKT

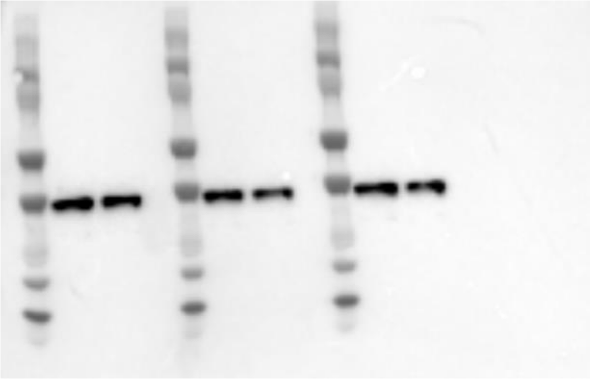

GAPDH

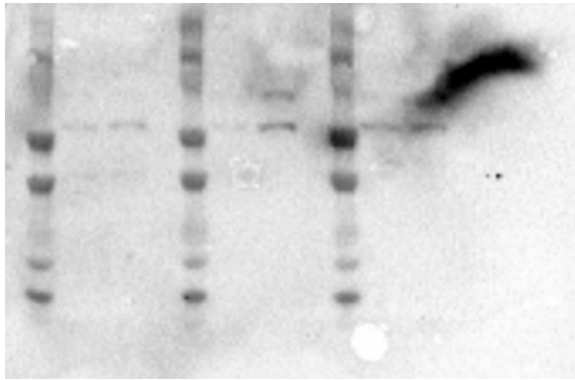

pAKT

OV8-10uM

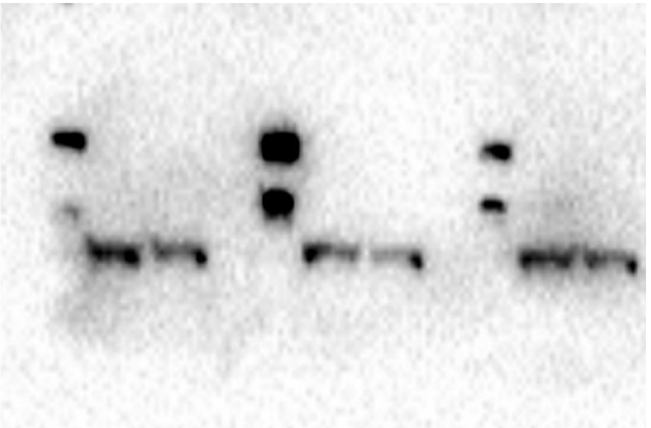

GPT2

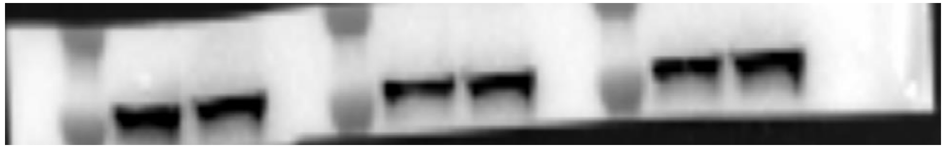

PSAT1

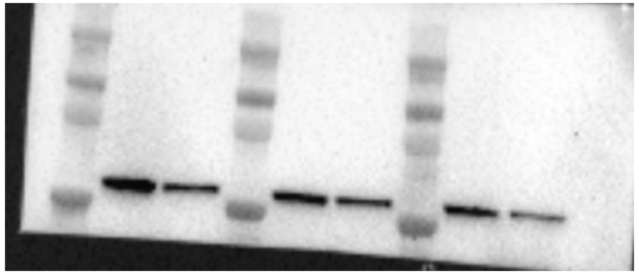

CEP55

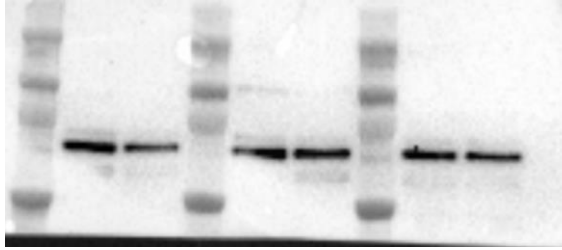

NumbL

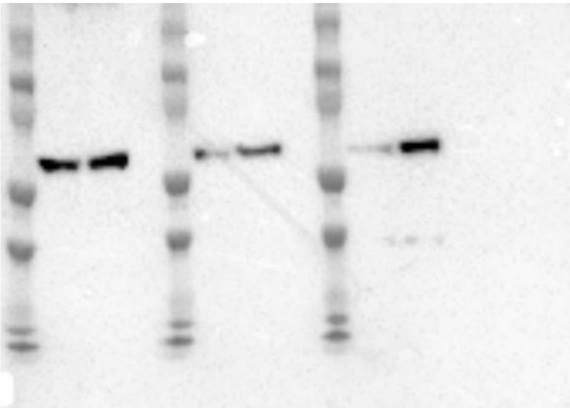

ASNS

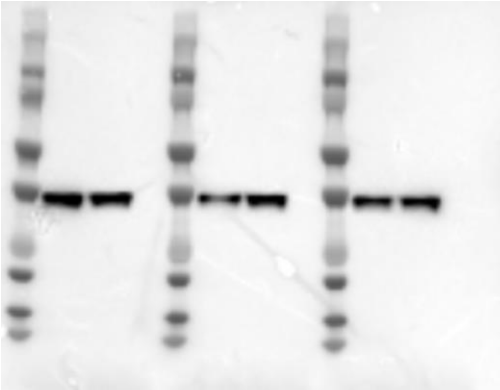

GAPDH

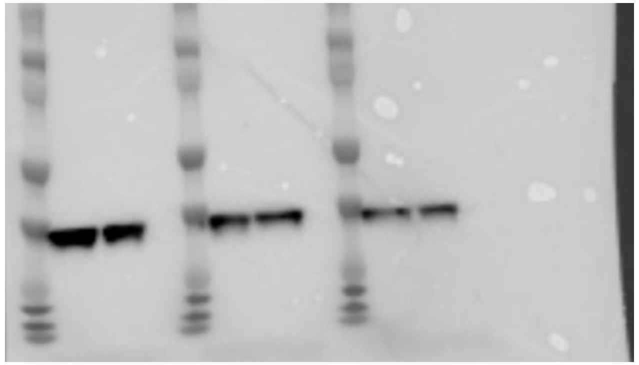

GAPDH

OV8-13.3uM

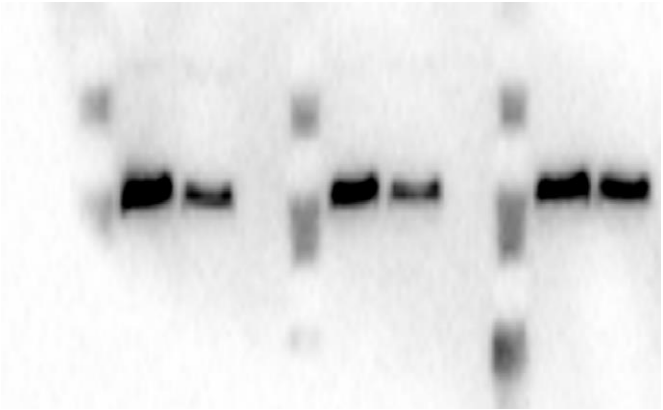

CEP55

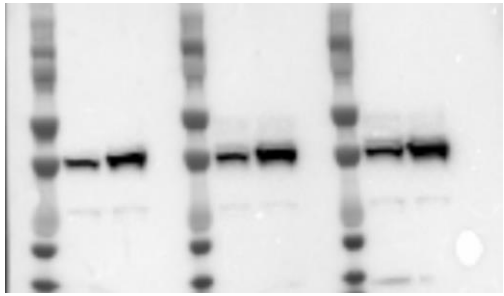

PSAT1

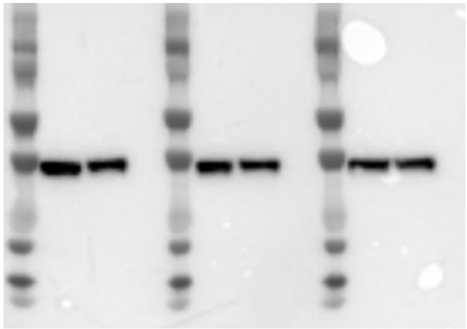

GAPDH

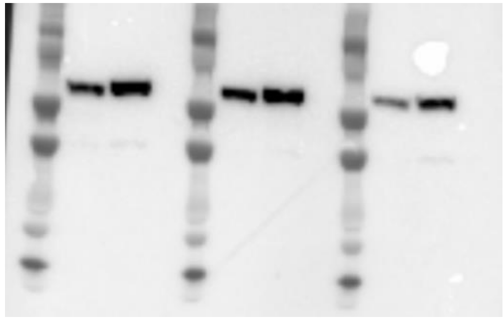

ASNS

PEA1

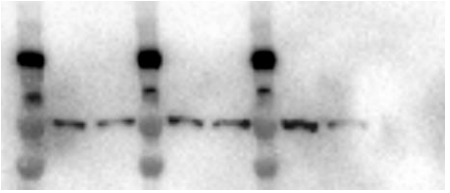

GPT2

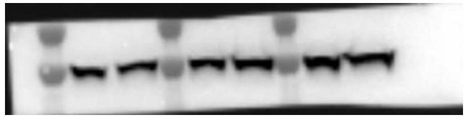

PSAT1

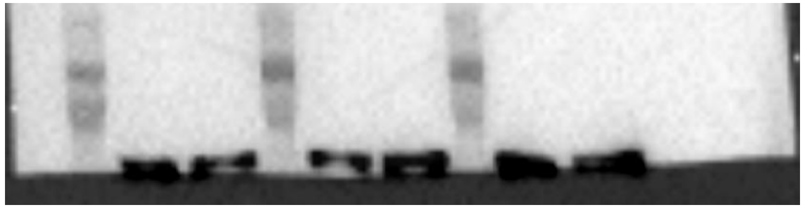

ASNS

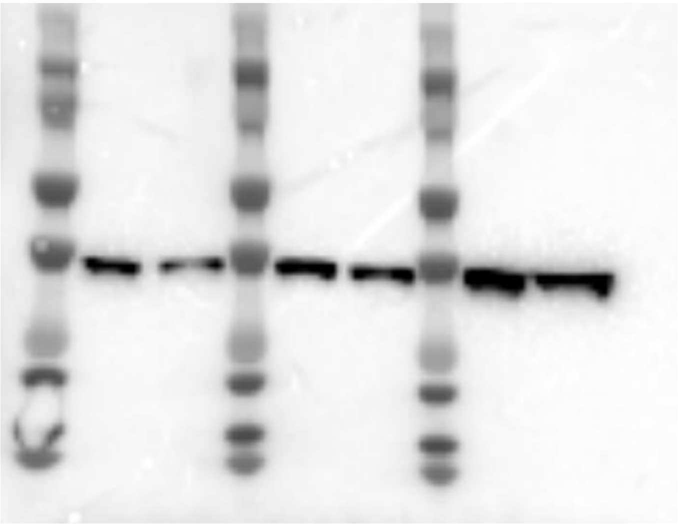

GAPDH

PEA2

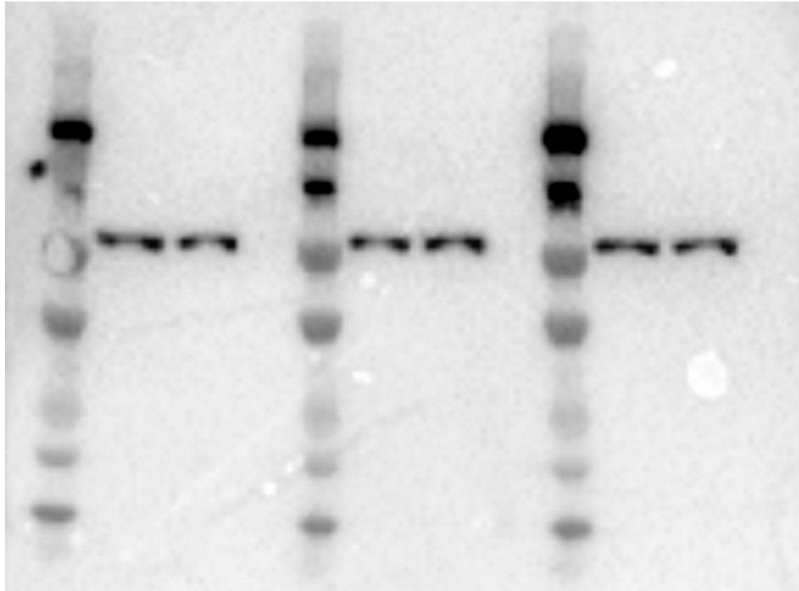

GPT2

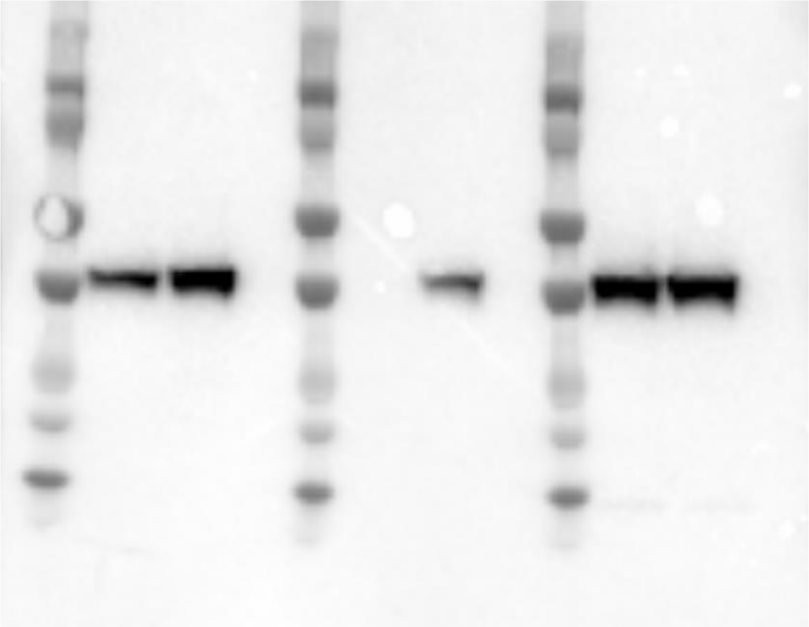

PSAT1

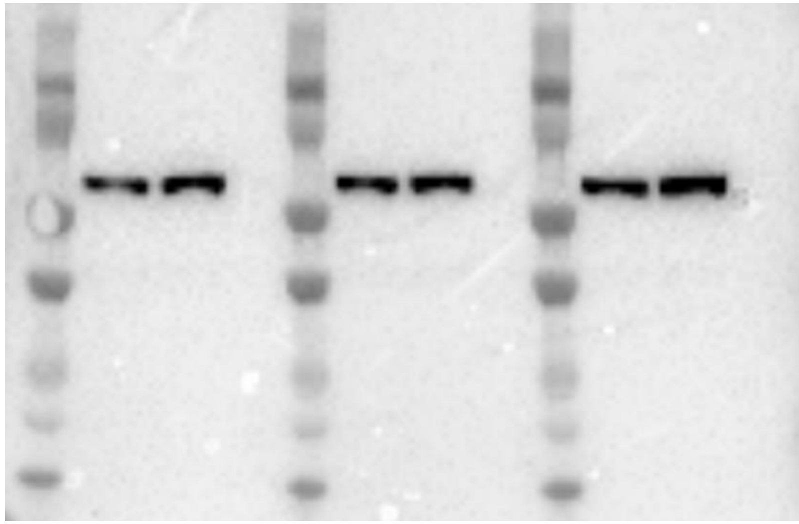

ASNS

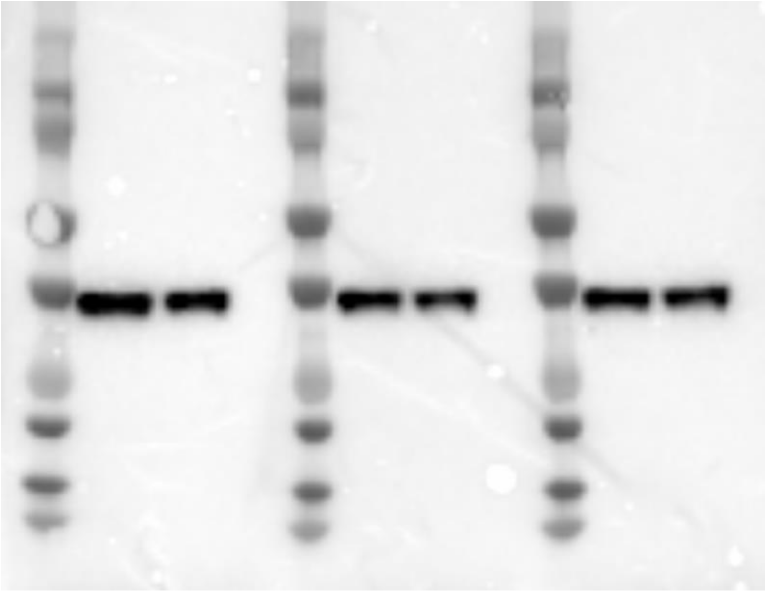

GAPDH
